# "Ierne"—An Appreciation

**Published:** 1918-12-21

**Authors:** 


### THE EDITOR'S LETTER-BOX—(*continued*).

### "Ierne"—An Appreciation.

*To the Editor of* The Hospital.

I don't know who "Ierne" is; I don't want to know. But whoever he is, he is a sensible man, and one of the greatest champions the trained nurse has ever had. I want him to know that some nurses appreciate him, and heartily endorse every proposal for reform which his articles contain. For myself, I cannot grumble. I am a trained nurse, and hold an important public post. I am well paid; I have short hours, long holidays, and I enjoy life as every educated and trained woman should; but my heart goes out to the hundreds of trained nurses all over the country who are hard worked, badly paid, and in many cases poorly housed and badly fed. It should not be so. Why don't all nurses join the College, and through the College demand that these things shall be altered? The nurses of the future must and shall be better paid. In the meantime, "Carry on," "Ierne."

Eileen.

